
JOURNAL INFORMATION
==============================
NLM Title Abbreviation: ScientificWorldJournal
Journal ID: ScientificWorldJournal
NLM Journal ID: 101131163
Journal ID: TSWJ

The Scientific World Journal

ISSN: 2356-6140
EISSN: 1537-744X
Publisher: Wiley

ARTICLE INFORMATION
==============================
PMCID: PMC6009225
PMID: 12920315
DOI: 10.1100/tsw.2002.874
Article ID: 160413
Article version: 1
Subjects: Editorial

Environmental Pollution in India: Boycott Plastic Bags

Manning William J. *
University of Massachusetts, Amherst, MA, USA
*William J. Manning: aamanning@mindspring.com
Publication date: 2002
Electronic publication date: 2002 Jul 4
Volume: 2
First page: 1874
Last page: 1875
Received 2002 Jun 7; Accepted 2002 Jun 7
Copyright: Copyright © 2002 William J. Manning.
Copyright year: 2002
License: This is an open access article distributed under the Creative Commons Attribution License, which permits unrestricted use, distribution, and reproduction in any medium, provided the original work is properly cited.
License URL: https://creativecommons.org/licenses/by/3.0/

Keywords: urbanization, traffic, exhaust gases, poverty, heavy metals, plastic bags

==============================
**Commentary**
*TheScientificWorldJOURNAL* (2002) 2, 1874–1875
ISSN 1537-744X; DOI 10.1100/tsw.2002.874

### Environmental Pollution in India: Boycott Plastic Bags

William J. Manning
*University of Massachusetts, Amherst, MA*

E-mail: aamanning@mindspring.com

*Received June 7, 2002; Accepted June 7, 2002; Published July 4, 2002*

---

**KEY WORDS:** urbanization, traffic, exhaust gases, poverty, heavy metals, plastic bags

**DOMAINS:** terrestrial environmental toxicology

---

India has more than a billion people in a land area smaller than China or the U.S. This has led to serious environmental pollution problems. During a recent visit to India, to participate in the Second International Conference on Plants and Environmental Pollution (ICPEP-2), I experienced some of these problems in places like Agra, Delhi, and Lucknow. Overpopulation, urbanization, poverty, and ignorance combine to cause air, soil, and water pollution and solid waste accumulation.

As in many other parts of the world, people from the country are migrating to cities and towns seeking jobs, housing, and food; in the process overwhelming the social infrastructure. In Lucknow, for example, dense groups of people live along the sides of the streets. The rhythm of daily life for them consists of finding enough food and fuel for the day to survive. Wood and dried manures fuel fires used for cooking and warmth at night. All of these fires release extensive smoke and particulates.

In the streets, there is incessant, dense, noisy traffic. Vehicles are so close together that you can reach out and touch the ones near you. This is a good incentive to keep your arms inside taxis at all times! My first few rides were terrifying and amazing as the horn-blowing drivers lurched and lunged forward through near-solid concentrations of bicycles, motor scooters, rickshaws, three-wheelers, cars, vans, buses, and trucks. Eventually I realized that the traffic moved continually forward in relative safety because alert, aggressive, and fearless drivers found creative nonviolent ways to find short-term gains and advantages. Road rage was not evident.

The best way to experience air pollution was to be out in the traffic. While travelling by rickshaw in Delhi, I was almost overwhelmed by diesel and gasoline exhaust fumes, containing partially burned hydrocarbons, oxides of nitrogen, fine particulates, sulfur compounds, and a host of other organic compounds. Few vehicles seemed to have emission control devices. The government claims that air pollution is decreasing, but this seems difficult to believe when you are out in the traffic.

©2002 with author.

Road dust was a problem everywhere, especially in Agra, near the Taj Mahal. Fortunately people like Professor D.K. Banerjee, Jawaharlal Nehru University, Delhi, are beginning to examine the extent and content of road dust. High concentrations of heavy metals, especially cadmium, are being found in road dusts.

Solid waste accumulation was evident in most places. Nonbiodegradeable plastic shopping bags seemed to be everywhere, often in great numbers. An article in the *Times of India* stated that millions of plastic bags are discarded every year. In addition to being a nuisance, they also contain inks and dyes that contain heavy metals, especially cadmium. An encouraging sign that the plastic bag problem is being taken seriously were the many billboards that I saw with the message: Boycott Plastic Bags. Opportunities to recycle plastic bags were not available.

Research on the nature and management of environmental pollution problems is hampered by ageing facilities and equipment, or lack of equipment, and lack of monitoring data. A hopeful sign for University researchers is an announcement by the government that it will double funding for infrastructure and laboratory instrumentation. Increased funding will also be applied to school libraries and the development of electronic databases. Hopefully some of this funding will find its way to environmental scientists!

At ICPEP-2, I talked with my colleague Professor Nigel Bell, Imperial College, London. As a frequent visitor to India, Professor Bell feels that there is an urgent need to create awareness among the general public about environmental problems in India. Mass education programs should start with school children, who could later educate their more tradition-bound parents. The Boycott Plastic Bags program might serve as a model for this awareness program.

### REFERENCE

Bagla, P. (2002) Academic science gets big boost in budget: India. *Science* **295,** 1993.

**This article should be referenced as follows:**

Manning, W.J. (2002) Environmental pollution in India: boycott plastic bags. *TheScientificWorldJOURNAL* **2**, 1874–1875.
